# Particleboards Bonded by an Imidazole-Based Adhesive System

**DOI:** 10.3390/ma16227201

**Published:** 2023-11-17

**Authors:** Alexander Scharf, Carmen-Mihaela Popescu, Henric Dernegård, Johan Oja, Graham Ormondroyd, Sergej Medved, Dick Sandberg, Dennis Jones

**Affiliations:** 1 Wood Science and Engineering, Luleå University of Technology, Forskargatan 1, SE-93187 Skellefteå, Sweden; dick.sandberg@ltu.se (D.S.); dennis.jones@ltu.se (D.J.); 2 Petru Poni Institute of Macromolecular Chemistry, 41A Grigore Ghica Voda Alley, 700487 Iasi, Romania; mihapop@icmpp.ro; 3 Holmen AB, Strandvägen 1, SE-11451 Stockholm, Sweden; henric.dernegard@holmen.com; 4 Norra Timber, Skeppargatan 1, SE-90403 Umeå, Sweden; johan.oja@norraskog.se; 5 Biocomposites Centre, Bangor University, Deiniol Road, Bangor LL57 2UW, UK; g.ormondroyd@bangor.ac.uk; 6 Biotechnical Faculty, University of Ljubljana, Jamnikarjeva 101, 1000 Ljubljana, Slovenia; sergej.medved@bf.uni-lj.si

**Keywords:** adhesion, citric acid, esterification, ionic liquids, wood composite

## Abstract

Particleboards with different combinations of the adhesive material imidazole, citric acid, and sorbitol were produced. Softwood sawdust from a Swedish sawmill was mixed with an aqueous solution of the chemicals and then dried to 0% moisture content prior to pressing. The boards were pressed to a target density of 700 kg m^−3^ at either 200 °C or 220 °C for 10 min. The hygroscopic and mechanical properties of the boards were clearly better at 220 °C than 200 °C for all used chemical combinations. A combination of imidazole (14.4 wt%) and citric acid (11.3 wt%) led to the best results, where the thickness swelling after 24 h of water immersion was 6.3% and the internal bonding strength was 0.57 MPa. The modulus of rupture and modulus of elasticity were 3.3 MPa and 1.1 GPa, respectively. Cyclic accelerated weathering showed exceptional stability with a thickness change after boiling and drying of only 2.1% compared to the initial dry thickness. This study indicates that the presence of imidazole leads to greatly improved hygroscopic properties and good internal bonding strength when used in particleboards.

## 1. Introduction

Particleboards are well-established wood composites commonly used in furniture and interior design as well as claddings. In 2022, the European production of particleboards (44 million m^3^) made up 42% of the global particleboard production, whilst Swedish production only accounted for just over 1% of the European production volume [1]. Particleboards are commonly manufactured from wood particles by either flat-pressing or extrusion methods, which allows the utilization of low-quality timber, small-diameter logs, residuals from the wood processing industries, and reclaimed timber [2,3]. This makes particleboards ecologically very important, by promoting sustainability and reducing waste [4]. Additionally, other lignocellulosic materials have been shown to be suitable for the production of particleboards [5,6].

The ecological and economical benefits of using lignocellulosic materials such as timber, bagasse, straw, or flax are reduced by the need for/use of synthetic resins as adhesives. These resins are usually thermosetting systems, typically involving a condensation reaction between formaldehyde and urea, melamine, phenol, or resorcinol [7]. Urea formaldehyde is the most commonly used adhesive system for particleboards used in dry conditions. Urea formaldehyde resin cures relatively quickly, with pressing times as low as 4 mm per second for medium-thickness particleboards [8] and temperatures of around 120 °C to 130 °C [9], resulting in efficient production and shorter manufacturing cycles. The drawback of urea-formaldehyde-bonded boards are their low moisture resistance and high levels of formaldehyde emission. Unreacted free formaldehyde exists in the boards, and additional formaldehyde is formed by moisture-induced reversed reactions during the service life of the product [8]. Formaldehyde is a volatile organic compound and can be released into the air over time, resulting in headaches, nose and throat irritation, and fatigue in occupants exposed to it over extended periods [10]. Formaldehyde is also listed as carcinogenic to humans and can cause certain types of cancer, e.g., cancer of the paranasal sinuses, nasal cavity, and nasopharynx [11]. The moisture resistance can be addressed by adding up to 20–25% of melamine to the urea-formaldehyde system [12], which also improves the load-bearing capacity of the material, most likely as a result of greater cross-linking within the resin matrix [13]. Another common approach is the application of phenol formaldehyde (PF) instead of urea formaldehyde, with these systems exhibiting high moisture resistance and low formaldehyde emissions. However, the use of PF has significant drawbacks; for instance, the slower curing time often necessitates higher press temperatures, and significant browning of the boards can occur [14]. Additionally, both melamine and PF-resins are more expensive than urea formaldehyde, whilst all three variants are synthetic materials derived from fossil fuels and have associated environmental impacts.

Regulations pertaining to formaldehyde emissions, future restrictions on fossil resources, and environmental considerations have prompted the search for green and formaldehyde-free adhesives. Various adhesive systems have been studied, including tannin [15,16], technical lignin [17,18], proteins [19], sucrose, and ammonium dihydrogen phosphate [20], to name a few. However, adhesive systems that are completely devoid of formaldehyde whilst meeting the technical requirements of current manufacturing practices (other than isocyanate-based adhesives) are scarce or non-existent [21].

In recent years, several researchers have turned their attention to citric acid as a bonding agent for wood composites. Citric acid, a readily available and low-cost chemical, is commonly found in citrus fruits and can be produced through microbial fermentation using *Aspergillus niger* [22]. Initially used in the cotton industry [23], citric acid and the structurally similar polycarboxylic acid were later adapted for wood modification [24,25]. The reaction mechanism between wood and citric acid involves a two-step esterification process, where a cyclic anhydride is initially formed and reacts with hydroxyl groups in the wood to create ester linkages [26]. Wood modification with citric acid offers several advantages, such as reduced water absorption, enhanced resistance against termites and fungi, an improved modulus of elasticity (MOE) and compression strength, and better dimensional stability [27]. However, it is important to note that there are some disadvantages, including a reduction in the modulus of rupture (MOR), increased brittleness, and yellowing of the treated wood [28].

The three carboxyl groups present in citric acid make it well-suited for cross-linking, as it allows for the easier formation of ester bonds compared to compounds with only two acid groups, such as maleic acid or succinic acid. This cross-linking ability between the carboxyl groups of citric acid and the hydroxyl groups of wood enables citric acid to be used as a binder in particleboards [29,30]. For instance, Umemura et al. [31] utilized 20 wt% citric acid at 200 °C in particleboards made from recycled wood particles, achieving a modulus of rupture (MOR) of 10.7 MPa, modulus of elasticity (MOE) of 3.3 GPa, and internal bonding strength (IB) of 0.32 MPa. However, the thickness swelling in water was found to be relatively high. Kusumah et al. [32] pressed particleboards from sweet sorghum bagasse with 20 wt% citric acid, finding 200 °C for 10 min to be the optimal pressing temperature. The bending properties of these boards were two-thirds of the values achieved with phenol formaldehyde resin, while the internal bonding strength (IB) and thickness swelling were slightly better in the boards bonded with citric acid. Similarly, Ferrandez-Garcia et al. [33] obtained comparable results when producing particleboards from giant reeds (*Arundo donax* L.) and citric acid. Huaxu et al. [34] bonded rubberwood particles with 10–20 wt% citric acid, observing inferior properties compared to urea formaldehyde resin; however, the properties improved with increasing citric acid concentration, and the resistance against termite and fungal attack improved drastically. The varying results in citric-acid-bonded particleboards discussed here are likely due to different pre-drying times of the particles used, as moisture content strongly influences the resulting properties [35].

**Figure 1:**
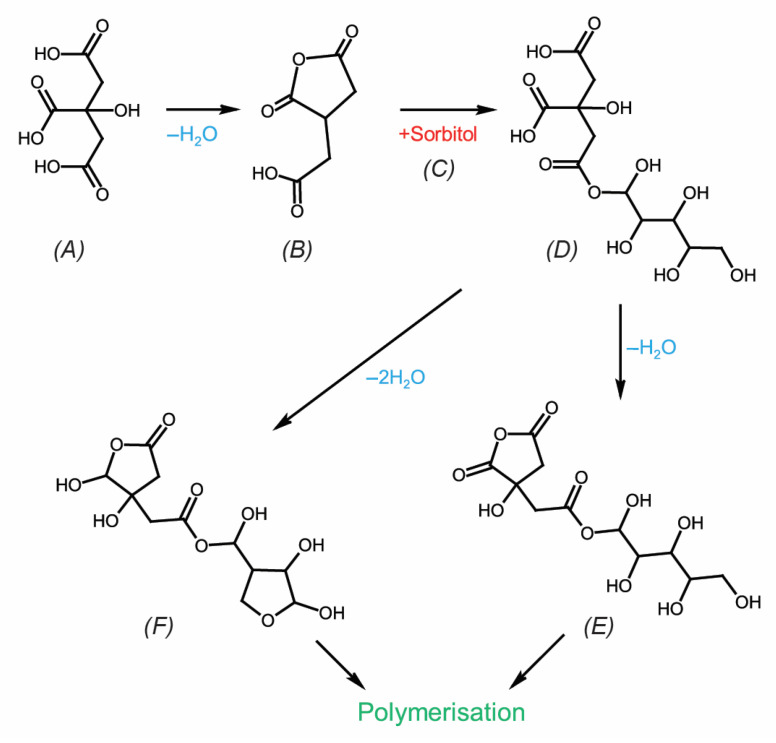
The reaction mechanism between (**A**) citric acid and (**C**) sorbitol. Intermediate (**B**,**E**) cyclic anhydride, (**D**) ester, and (**F**) anhydrosorbitol ring. Adapted from [41].

The performance of citric-acid (CA)-treated wood can be enhanced by incorporating compounds containing alcoholic hydroxyl groups such as glucose [36,37], sucrose [31], glycerol [38,39,40], and sorbitol [41,42,43]. These compounds react with the hydroxyl groups, leading to polymerization and increased chemical fixation. Umemura et al. [31] utilized a combination of citric acid and sucrose at a resin content of 30 wt% with a 1:3 ratio of CA to sucrose to produce particleboards at 200 °C for 10 min; the resulting boards exhibited high bond strength and a thickness swelling of 12% after 24 h of water exposure. The polymerization of citric acid and sorbitol in an aqueous solution was initially demonstrated in a patent by Centonella and Razor [44]. Subsequent work by Doll et al. [45] polymerized the same compounds at different molar ratios in a vacuum oven. The authors found that the reaction was more rapid at 150 °C than at 110 °C and that using an equal or lower molar ratio of acid to hydroxyl groups (3:1 CA:sorbitol) resulted in partially insoluble polymers; they proposed the formation of intermediates and subsequently a citrate sorbitol ester, as shown in Figure 1. Kiljunen et al. [46] applied a wood modification process using an aqueous solution of 5% citric acid and 10% alcohol as well as a 2% sodium hypophosphite catalyst, resulting in a 50% leaching resistance. Larnøy et al. [41] reported the polyesterification of citric acid and sorbitol in a 3:1 molar ratio for solid Scots pine (*Pinus sylvestris* L.) wood at 140 °C for 18 h. Leaching tests indicated excellent leaching and fungal resistance, as higher curing temperatures led to higher conversion rates of functional groups, resulting in a denser crosslinked networks in the cell wall. Parallel work [47] showed the suitability of citric acid-sorbitol for the treatment of European beech (*Fagus sylvatica* L.). Lin et al. [43] applied the same system to particleboard production and demonstrated its suitability as an alternative adhesive. The best results were obtained at a curing temperature of 200 °C for 13 min, utilizing in-situ polyesterification of CA and sorbitol as the binding agent. At a resin content of 10 wt%, the particleboards achieved a thickness swelling of 40%. For more detailed information on citric acid in wood modification and wood binding, readers are referred to the review by Lee et al. [48].

An alternative option for environmentally friendly binding agents in the production of particleboard is the use of ionic liquids (ILs). These unique solvent systems have gained attention as potential binders in wood composites due to their distinctive properties and environmental advantages [49]. ILs offer several benefits, such as low volatility, customizable properties, the ability to dissolve and plastify a wide range of substances (including polymers and lignocellulosic materials) [50,51], and an increasing popularity in biorefining processing [52]. Ionic liquids, which are liquid-state salts at or near room temperature, consist typically of large organic cations paired with small inorganic or organic anions. They exhibit negligible vapor pressure, high thermal stability, and excellent solvating capabilities, making them suitable for dissolving and modifying various components of various natural materials, including wood. By dissolving lignin, cellulose, and hemicellulose, ILs enable the extraction and separation of these components from lignocellulosic biomass, which can then be utilized as building blocks for adhesives or directly incorporated into the particleboard formulation. Promisingly, ILs have shown potential for enhancing the mechanical properties, water resistance, and dimensional stability of particleboards. Orelma et al. [53] successfully bonded sawdust using the ionic liquid 1-Ethyl-3-methylimidazolium acetate in a two-step process involving 3 h of pressing followed by 24 h of extraction in methanol. The resulting board exhibited compatibility with commercial particleboards, although achieving good results required a high proportion of ionic liquid. However, this process is time-intensive and requires expensive chemicals in large quantities, rendering it economically impractical at present.

Imidazolium is a commonly found cation in ionic liquids. In an unpublished attempt to synthesize an imidazolium-containing ionic liquid in-situ during particleboard production, imidazole was utilized. Interestingly, particleboards pressed with imidazole exhibited high hygroscopic stability. Imidazole, a five-membered ring compound comprising two nitrogen and three carbon atoms, possesses acidic and basic properties due to the presence of two non-adjacent nitrogen atoms. Imidazole and its derivatives have demonstrated various properties, including antifungal [54], anti-inflammatory [55], and antimicrobial effects [56], making it a commonly employed ingredient in drug synthesis [57]. Imidazole has been used as a reagent in the production of nanocellulose [58,59], in combination with thermoplastic starch and wood fibers [60], and as a wood modification agent [61]. To the best of our knowledge, there are no other previous reports on the use of imidazole as a binder in wood composites.

The objective of this study was to explore formaldehyde-free bonding systems for wood composites using imidazole in combination with citric acid and sorbitol. Wood particles were sprayed with solutions containing various combinations of imidazole, citric acid, and sorbitol and then pressed in a dry state at different temperatures.

## 2. Materials and Methods

### 2.1. Materials

Wood processing residuals in the form of sawdust were obtained from a sawmill in northern Sweden (Holmen AB, Kroksjön, Västerbotten, Sweden), processing Scots pine (*Pinus sylvestris* L.) and Norway spruce (*Picea abies* (L.) Karst.). The green sawdust was spread out and dried in an oven at 60 °C for 48 h. A sieve with apertures of 3.2 mm in the largest dimension was used to remove large particles, and the fraction passing through the sieve was used for particleboard preparation. The particulate size was in the range of approx. 0.25–2.5 mm.

Combinations of >98% synthesis-grade imidazole (C_3_N_2_H_4_) powder (IoLiTec Ionic Liquids Technologies GmbH, Heilbronn, Germany), 99.9% analytical-grade citric acid (C_6_H_8_O_7_), and ≥96% technical-grade D (-)-sorbitol (C_6_H_14_O_6_) powder (VWR International AB, Stockholm, Sweden) were dissolved in deionised water to produce solutions with a 10 wt% solid content. As a reference, a UF-resin (TS Resins Ltd., Mold, UK) with a pH value of 8.5 at 25 °C, a viscosity of 250 cP at 25 °C, and a solid content of 63.5% was used. The combinations used are shown in Table 1.

### 2.2. Specimen Preparation

Prior to board manufacture, the dried particles and respective solutions were combined in a rotating vessel by spraying to a target wt% chemical content relative to the particle mass (Table 1). The particle–chemical mixtures were dried in an oven at 70 °C for 24 h to evaporate most of the water. The mixtures were divided into equal parts and three specimens were produced per group. The dried mat was pressed as a single-layer board in an open system hydraulic press (HLOP15, Höfer Presstechnik GmbH, Taiskirchen, Germany) using a mould of size 220 mm × 220 mm. The mats were pressed for 10 min at a temperature of 200 °C or 220 °C to a target thickness of 8 mm and secured by mechanical stops, to achieve a theoretical target density of 700 kg m^−3^. The reference group UF180 was prepared at the BioComposites Centre (Bangor University, Bangor, Wales). The urea formaldehyde content was 12.5 wt% and boards were pressed at a temperature of 180 °C for 4 min. After pressing and cooling, the particleboards were weighted to calculate mass loss during pressing.

### 2.3. Specimen Testing

The pressed particleboards were separated into different specimens to determine hygroscopic and mechanical properties. Each property was determined on 6 replicas per group, with 2 replicas taken from each board. After cutting but prior to further testing, all specimens were conditioned at 20 °C and 65% relative humidity for 3 weeks; we measured mass and thickness after conditioning, to determine the equilibrium moisture content (EMC). Density profiles of the conditioned specimens of size 50 mm × 50 mm were obtained by X-ray densitometry with a laboratory DP analyser (DENSE-LAB X, Electronic Wood System GmbH, Hameln, Germany) with a spatial resolution of the X-ray beam of 50 μm. Measurements were undertaken with step intervals of 44 μm in the thickness direction. 

Thickness swelling (TS) and water uptake after 2 h and 24 h immersion in water were tested on 50 mm × 50 mm × 8 mm specimens according to the EN 317 standard [62]. A cyclic accelerated ageing treatment was utilized on the same specimens, in which the specimens were dried at 103 °C for 12 h, immersed in 70 °C warm water for 24 h, dried at 103 °C for 12 h, boiled in water for 4 h, and dried at 103 °C for 12 h. Thickness and mass changes were recorded at each step. This approach is similar to the procedure used by Umemura et al. [31].

The internal bonding strength was determined on 50 mm × 50 mm × 8 mm specimens according to the EN 319 standard [63]. The modulus of elasticity (MOE) and the modulus of rupture (MOR) in bending were tested in conjunction in a three-point bending test on specimens of size 220 mm × 50 mm × 8 mm according to the EN 310 standard [64]. Specimens were loaded in an MTS Criterion Series 40 universal testing machine (MTS System Corporation, Eden Prairie, MN, USA) equipped with a 10 kN load cell for the IB test and a 500 N load cell for the bending test.

The measurements of TS, IB, MOR, and MOE were compared to the requirements for the board types in humid conditions P3, P5, and P7 according to the EN 312 standard [65].

### 2.4. Chemical Analysis

Infrared spectra of the boards were measured with an Alpha Bruker FTIR spectrometer (Bruker Optics, Ettlingen, Germany) in transmittance mode using KBr pellets. Before measurement, the samples were powdered using ball milling equipment. The concentration of the sample in the pellet was 3 mg/300 mg of KBr. The spectra were recorded in the 4000–400 cm^−1^ region, at a resolution of 4 cm^−1^. The spectra presented in this study are the average spectrum over 5 repeated recordings of the same sample.

Processing was performed using the OPUS 7.5 program and Origin 2023b.

## 3. Results and Discussion

### 3.1. Mechanical and Hygroscopic Properties

**Figure 2:**
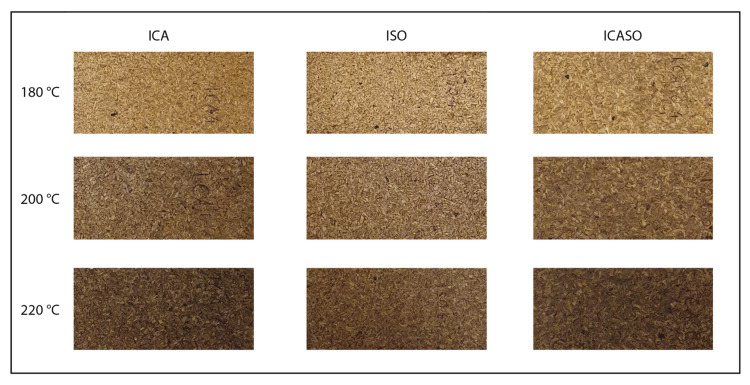
The typical appearance of boards pressed at 180 °C, 200 °C, and 220 °C.

The degree of browning due to heat exposure and the presence of chemicals during pressing is shown in Figure 2. The pressed boards which contained imidazole were either brown (200 °C) or dark brown (220 °C) in colour, while the colours of the boards with citric acid only stayed close to the original colour of the used sawdust. Browning of the imidazole-containing boards, as well as changes in the mechanical and hygroscopic properties, were significantly lower for boards pressed at 180 °C and below in a pre-test. At 180 °C, the TS was 20.0–55.2% and IB was 0.05–0.25 MPa among the groups; this does not meet any requirements for particleboard classes according to EN 312 [65]. This indicates that the targeted chemical reactions take place at 200 °C and above; therefore, this study covers only the results obtained at 200 °C and 220 °C.

Table 2 shows the weight loss after pressing, density at EMC, EMC, and the thickness swelling from dry to equilibrium state, respectively. Weight loss during pressing was higher at 220 °C than at 200 °C, with a maximum loss of 14.3% when imidazole and citric acid were used in combination. For citric acid, the weight loss could be attributed to the ester linkage of the carboxyl groups of the citric acid to the hydroxyl groups of the wood [26], producing water as a by-product which evaporated during the pressing process. The bonding mechanism and potential by-products of the reaction between imidazole and citric acid/wood are unknown. It is possible that imidazole interacts mainly with the lignin, depolymerizing the lignin into compounds such as vanillin and vanillic acid [58]. This was supported by testing combinations of imidazole and delignified fibres, which resulted in boards too fragile to be handled.

The EMC was similar in all groups, with boards pressed at 220 °C showing a slightly lower EMC than those pressed at 200 °C, most likely due to thermal degradation of the hydroxyl groups of the hemicelluloses. The effect of chemical treatment and pressing temperature was more pronounced for the thickness swelling from dry state to EMC. Both imidazole and citric acid showed a positive influence on this property, being more stable at higher pressing temperatures. The effect was noted to be slightly larger when the two chemicals were combined. Alternatively, the addition of sorbitol to imidazole had a negative effect on the thickness swelling, likely due to the large number of available hydroxyl groups in sorbitol.

**Figure 3:**
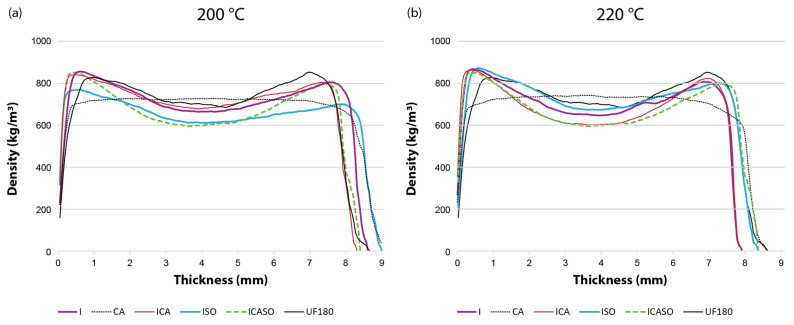
Mean density profiles for particleboards pressed at (**a**) 200 °C and (**b**) 220 °C and the reference group UF180. Number of specimens per group = 12.

Mean density profiles of the conditioned boards are shown in Figure 3. All groups except CA200/220 exhibited “U”-shaped density profiles typical for particleboards. The flat density profiles in the citric acid-only treated boards indicated pre-curing of the chemical during pressing.

**Figure 4:**
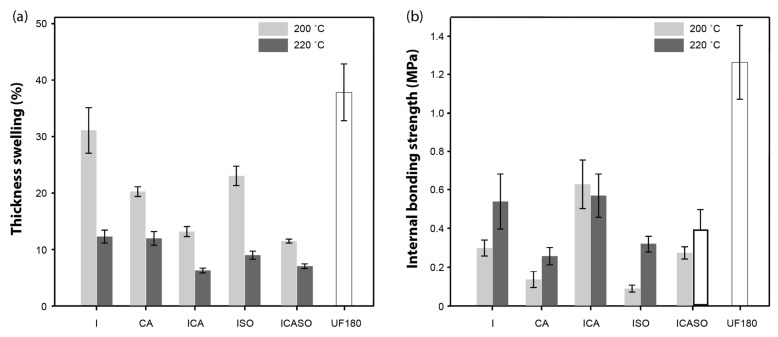
Mean ± standard deviation of (**a**) thickness swelling after 24 h immersion in water at 20 °C and (**b**) internal bonding strength of the conditioned boards pressed at 200 °C and 220 °C. Number of specimens per group = 6.

The thickness swelling (TS) after water soaking is shown in Figure 4a. Thickness swelling was greatly reduced in all groups when pressing at 220 °C compared to at 200 °C. This may be explained by different temperature-dependent mechanisms, such as thermal degradation of the boards and reactions of the used chemicals. I220 and CA220 showed TS values of 12.4% and 12.0%, respectively. However, in the combination, the TS was reduced to 6.3%. Similar results were achieved with a combination of imidazole, CA, and sorbitol. This may be explained by the higher total chemical loading in ICA and ICASO boards compared to imidazole alone. The reduced TS could be attributed to the polyesterfication of CA and sorbitol [41] and the impact of imidazole. The mechanism of imidazole is currently not known, though the presence of imidazole could lead to increased thermal modification, cross-linking to wood compounds, or the formation of lignin-derived compounds which might further interact with the cell wall polymers and introduced chemicals.

Figure 4b shows the internal bonding strength (IB). The use of imidazole at 220 °C led to a good IB while CA alone exhibited rather low values. The addition of citric acid to imidazole ICA200/220 led to slightly better IB, especially at a pressing temperature of 200 °C. The addition of sorbitol to imidazole reduced the IB drastically. The same was observed for the combination of all three chemicals (ICASO). This could be due to the hygroscopicity of unreacted sorbitol. These results indicate that a combination of imidazole and citric acid may involve interactions between the chemicals or their reaction products. One potential mechanism could be an imidazole-promoted bonding of phenolic hydroxyl groups of lignin to citric acid [30] or the reaction of the intermediate anhydride from citric acid with imidazole to produce N-acylimidazole.

**Figure 5:**
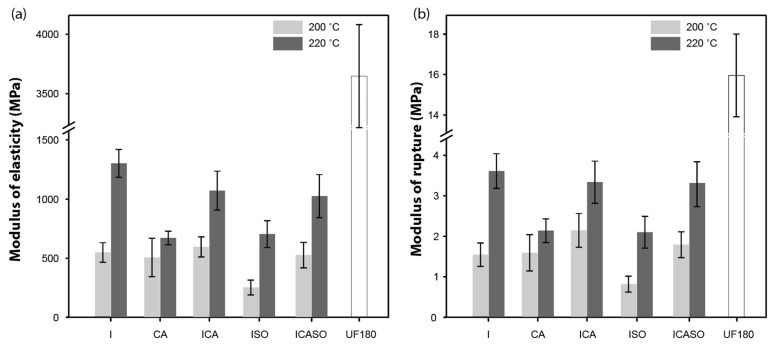
Mean ± standard deviation of (**a**) MOE and (**b**) MOR during bending of the conditioned boards pressed at 200 °C and 220 °C. Number of specimens per group = 6.

Figure 5 shows the MOE and MOR values of the boards. While some combinations were shown to be superior to the UF-control in TS and IB, this was not the case for the bending properties. A MOR of 3–4 MPa was achieved at a pressing temperature of 220 °C, with little variation between the groups. Only the groups CA220 and ISO220 showed even lower MOR values. The MOR was much lower in all groups compared to UF180 (16 MPa). A similar pattern between the groups was observed for the MOE. However, the difference between the treatment groups (1–1.5 GPa) and UF-control (3.6 GPa) was less extreme than in the MOR. The low bending properties coupled with relatively high IB could be explained by the different exposure times of the surface and core layer to the high pressing temperature. The surface layer may have undergone increased thermal and chemical-induced degradation, leading to strongly reduced mechanical properties (especially MOR); meanwhile, the particles in the core of the board were less affected and thus led to a relatively high IB [66].

**Figure 6:**
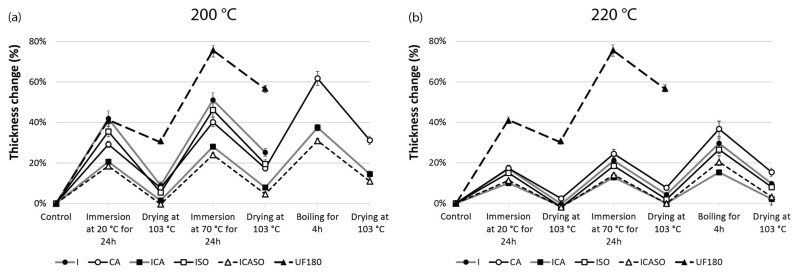
Mean thickness changes over cyclic accelerated ageing treatment for boards pressed at (**a**) 200 °C and (**b**) 220 °C. The reference thickness (control) is the dry thickness after pressing of the boards. Error bars indicate standard deviations. Number of specimens per group = 6.

Figure 6 shows the thickness swelling in the accelerated ageing treatment. At 200 °C the groups I and ISO—and to some extent CA—disintegrated during the boiling tests. The thickness swelling after the complete procedure was only 31% in CA200 and below 15% in ICA and ICASO, which is remarkable. At 220 °C, not only was disintegration almost absent but the achieved swelling values were exceptionally low. The thickness change in the dry state after boiling was 2% in ICA220 and 3% in ICASO220. Similarly, the CA boards showed very low values.

The mass loss due to leaching during the accelerated ageing treatment was always lower at 220 °C. CA220 showed a very low leaching of 2.4% in the first two cycles but partially disintegrated in the boiling step. For the remaining groups, the mass loss after the initial 24 h immersion in water was 6–8% in I, ICA, and ICASO and 11% in ISO. This grouping remained the same throughout the cyclic accelerated ageing process. After the boiling test, ICA220 exhibited a total mass loss of 16%, which is slightly lower than the UF-control at 21%.

**Figure 7:**
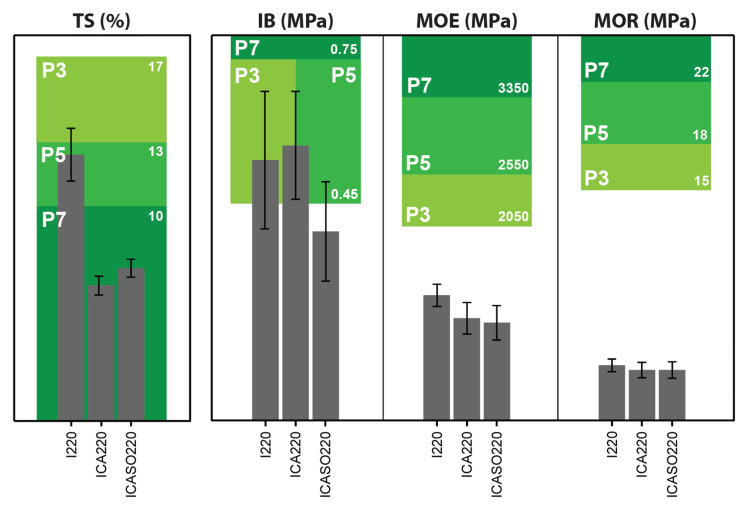
Comparison of thickness swelling (TS), internal bonding strength (IB), modulus of elasticity (MOE), and modulus of rupture (MOR) for three groups, compared with the respective requirements for the particleboard classes P3, P5, and P7 according to the EN 312 standard [65].

Figure 7 provides an overview of the discussed results in comparison with the requirements for particleboards in humid conditions. Even though high P-classes could be reached in terms of TS and IB, the bending properties were insufficient. Optimization of the raw materials and pressing parameters should be performed. Different chemical loadings and particle sizes between the surface and core layer as well as shorter pressing times (with increased temperature diffusion by means of applying steam in the core) could potentially negate detrimental effects and improve the bending properties.

### 3.2. Chemical Analysis

Structural features of the prepared boards were evaluated using infrared spectroscopy. The spectra of the particleboards were compared with the spectrum of the wood used as raw material in the boards.

**Figure 8:**
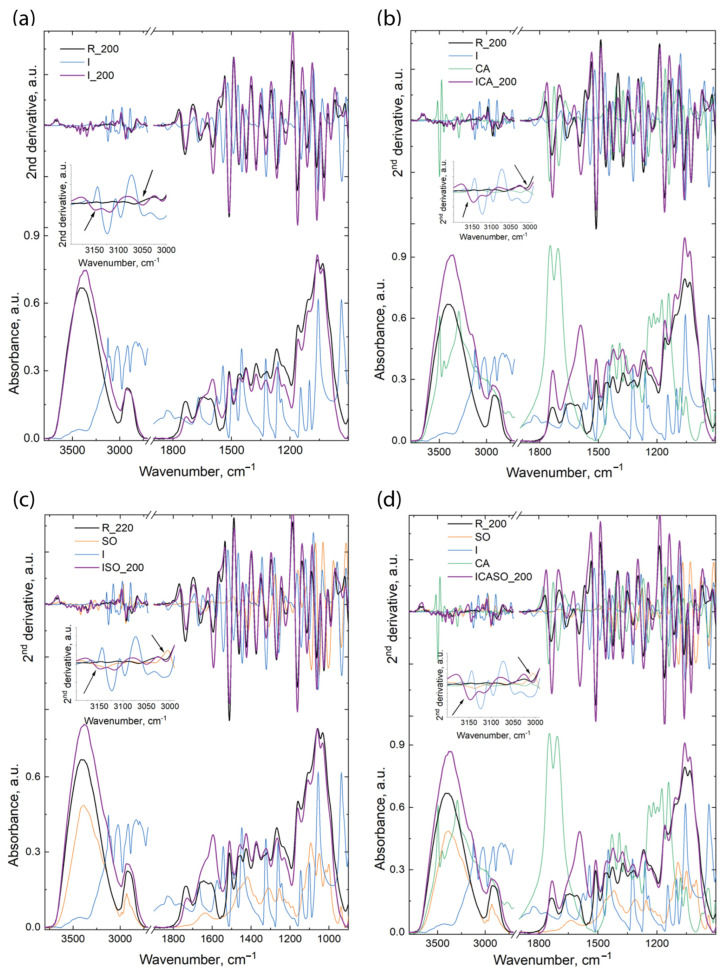
Infrared spectra and their second derivatives for the components and boards pressed at 200 °C for: (**a**)—I board and component spectra, (**b**)—ICA board and component spectra, (**c**)—ISO board and component spectra and (**d**)—ICASO board and component spectra.

**Figure 9:**
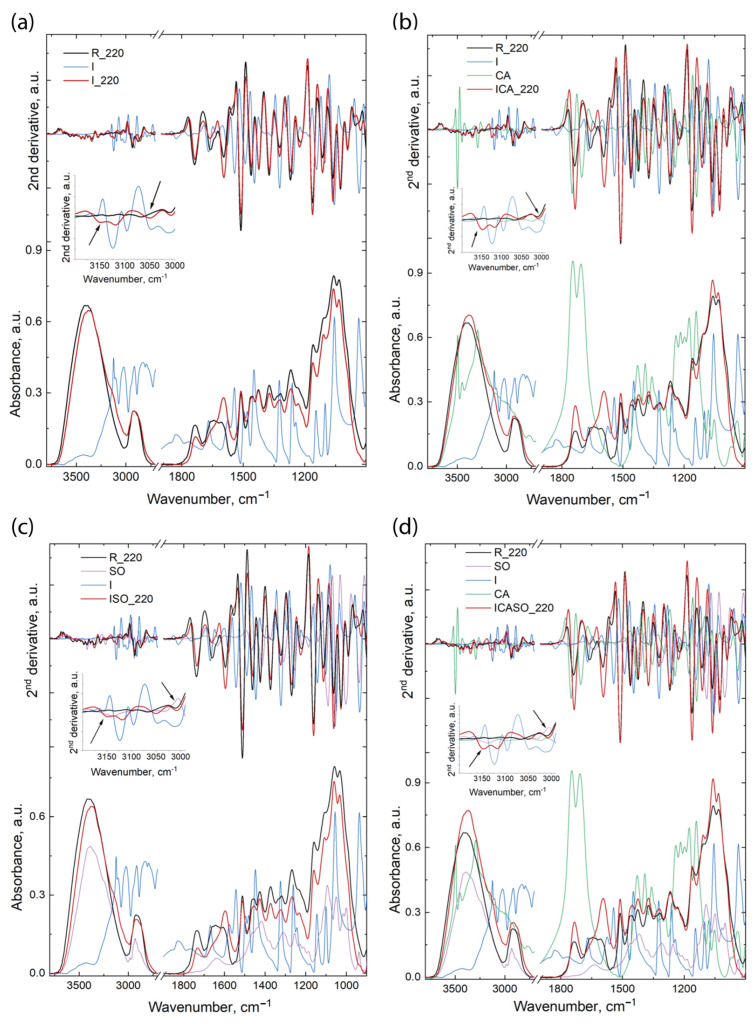
Infrared spectra and their second derivatives for the components and boards pressed at 220 °C for: (**a**)—I board and component spectra, (**b**)—ICA board and component spectra, (**c**)—ISO board and component spectra and (**d**)—ICASO board and component spectra.

Generally, the spectra of the wooden material presented two main regions: 3800–2700 cm^−1^ and 1800–400 cm^−1^. Moreover, wood and the other chemicals used for the preparation of the boards present many overlapped bands; hence, for a better evaluation of the differences which might appear in the components’ structures, as well as any possible interactions between the wood components and adhesives, the second derivatives of the spectra were calculated. Both the spectra and their second derivatives are presented in Figure 8 and Figure 9.

The modifications which appear in the spectra and their derivatives are independent of temperature. The differences between the boards pressed at 200 °C and 220 °C are related to further shifting of the bands or to a slight increase/decrease in their intensities; hence, we will evaluate only the variations appearing in the boards as compared against the wood materials and the pure chemicals, without indicating the temperature used for the pressing of the boards. The wood material was also treated under the same conditions as the boards, in order to avoid misinterpretation of the results (due to possible differences appearing in the spectra of wood during modification at high temperature). Similar to the boards, there was no large variation between the spectra of the wood material treated at 200 °C and the one treated at 220 °C. All the band maxima were taken from the second derivative spectra.

The spectra of the I board present differences in the 3750–2700 cm^−1^ region, as follows: the bands at 3347, 3285, and 3231 cm^−1^ increase in intensity in the I board spectra compared to the R spectrum. These bands are assigned to O(3)H…O(5) intramolecular hydrogen bonds as well as to O(6)H…O(3) intermolecular hydrogen bonds in cellulose [67,68]. The modification of the intensity of these bands indicates the presence of hydrogen bonds between the O-H groups from wood and N-H and N from the imidazole molecule. Further modifications were observed for the bands mainly assigned to N-H groups from the imidazole molecule, as well as to C-H from the methyl and methylene groups from both materials; thus, the bands at 3149, 3118, and 3007 cm^−1^ are shifted to lower wavenumbers (from 3156, 3123, and 3013 cm^−1^ in the I spectrum and 3011 cm^−1^ in the R spectrum); the band at 3050 cm^−1^ is shifted to higher wavenumbers from 3045 cm^−1^ (in the I spectrum). In the other region, differences are observed mainly for the bands at 1736 cm^−1^, which slightly decreases in intensity compared to R (reference wood) and is shifted to lower wavenumbers by 4 cm^−1^, and at 1595 cm^−1^, which increases in intensity in the I board spectra compared to the R spectrum. This later band is assigned to the C=C stretching vibration in the aromatic ring from lignin; however, because the other band at 1510 cm^−1^ (which also is a reference band for C=C in the aromatic ring vibration) is not modified in the same way, this band may also be assigned to conjugated C-O stretching vibrations and to C=C from imidazolium ions (proton conjugating system) [69]. Further, from the second derivative spectra, we observe that the bands located at 1257 cm^−1^ in the I spectrum and at 1269 cm^−1^ in the R spectrum appear at 1267 cm^−1^ in the board spectra, and the bands at 1234 cm^−1^ in the I spectrum and 1221 cm^−1^ in the R spectrum appear in the board spectra at 1225 cm^−1^. Moreover, the bands at 1323, 1162, and 1112 cm^−1^ only increased in intensity. All these modifications indicate the presence of imidazolium ions in the system.

When citric acid was added to the system (the ICA pressed boards (Figure 8b and Figure 9b)), the modifications were related to the bands at 3420 and 3347 cm^−1^, which increased in intensity in the ICA spectra compared to the R spectrum; at 3384 cm^−1^, which was shifted to 3382 cm^−1^ and slightly decreased in intensity; at 3286 and 3228 cm^−1^, which increased in intensity and were shifted to 3282 and 3232 cm^−1^ in the ICA spectra when compared to the R spectrum; and 3148 and 3117 cm^−1^ (in the ICA spectra), which were shifted to 3156 and 3123 cm^−1^ (in the I spectrum). All these bands are assigned to different inter- and intramolecular hydrogen bonds in cellulose, and their modification indicates the presence of hydrogen bonds between the N-H…O and N…O-H groups. Further modifications are related to the bands assigned to CH and NH bonds, as follows: the band at 3051 cm^−1^ in the ICA spectra is a combination of the bands from the I and CA spectra at 3045 cm^−1^ (in the I spectrum) and at 3061 cm^−1^ (in the CA spectrum); meanwhile, the band at 3008 cm^−1^ in the ICA spectra is shifted from 3013 cm^−1^ in the I spectrum and from 3011 cm^−1^ in the R spectrum.

In the fingerprint region, the main differences appear for the bands at 1735 cm^−1^, which increase in intensity compared to similar bands from the wood (R) spectrum, and at 1590 cm^−1^ in the ICA spectra, which increases in intensity and is shifted from 1596 cm^−1^ (in the R spectrum). The increase in the intensity of these bands indicates the presence of imidazolium ions in the system, as well as new C-O and C=O bonds appearing due to the presence of citric acid and the formation of new carbonyl bonds in the system. Further modifications were observed for the bands at 1269 cm^−1^ (in the ICA spectra), which is derived from the bands at 1257 cm^−1^ in the I spectrum, 1244 cm^−1^ in the CA spectrum, and 1269 cm^−1^ in the R spectrum, and at 1227 cm^−1^ in the ICA spectra, which is a band formed from the bands at 1234 cm^−1^ in the I spectrum, 1221 cm^−1^ in the R spectrum, and 1210 cm^−1^ in the CA spectrum. Other bands such as 1322, 1161, 1063, and 1025 cm^−1^ did not change their position but increased in intensity in the ICA spectra compared to the R spectrum. ISO board spectra (Figure 8c and Figure 9c) differences, as compared to the R, I, or SO spectra, appear for the following bands: 3417 cm^−1^ (in the ISO spectra) is shifted from 3420 cm^−1^ (in the R spectrum); 3346 and 3286 cm^−1^ increase in intensity in the ISO spectra compared to the R spectrum, while the band at 3234 cm^−1^ (in the ISO spectra) is shifted from 3229 cm^−1^ (in the R spectrum); 3150 cm^−1^ (in ISO spectra) is shifted from 3156 cm^−1^ (in the I spectrum) and 3136 cm^−1^ (in the SO spectrum); 3117 cm^−1^ (in the ISO spectra) is shifted from 3123 cm^−1^ (in the I spectrum); 3050 cm^−1^ (in the ISO spectra) is shifted from 3045 and 3061 cm^−1^ (in the I and SO spectra, respectively), while the band at 3007 cm^−1^ (in the ISO spectra) is shifted from 3013 and 3011 cm^−1^ (in the I and R spectra, respectively). In the fingerprint region, the bands at 1732, 1372, and 1268 cm^−1^ decrease in intensity in the ISO spectra compared to the R spectrum, while the bands at 1595, 1322, 1162, and 1026 cm^−1^ increase in intensity in the ISO spectra compared to the R spectrum and the bands at 1268/1225 cm^−1^ (in the ISO spectra) are shifted from 1257/1234, 1254/1218, and 1269/1221 cm^−1^ (in the I, SO, and R spectra, respectively).

The spectra and their second derivatives of the boards which use as adhesive all three components mentioned above (imidazole, sorbitol, and citric acid) and all components are presented in Figure 8d and Figure 9d. The differences between the bands from the pressed boards and components are as follows: the band at 3418 cm^−1^ (in the ICASO spectra) is shifted from 3420 cm^−1^ (in the R spectrum); the band at 3381 cm^−1^ (in the ICASO spectra) is shifted from 3385 cm^−1^ (in the R spectrum) and is slightly increased in width; and the bands at 3345, 3285, 3231, 3148, and 3118 cm^−1^ increase in intensity compared to the R spectrum. Moreover, the latter two bands also show shifts of the bands at 3156 and 3136 cm^−1^ in the I and SO spectra, as well as at 3123 cm^−1^ in the I spectrum, respectively. The bands at 3049 and 3006 cm^−1^ are shifted in the ICASO spectra compared to the bands at 3045 and 3013 cm^−1^ (in the I spectrum), 3061 cm^−1^ (in the SO spectrum), and 3011 cm^−1^ (in the R spectrum). Further, the bands at 1735, 1592, 1322, 1228, 1162, and 1026 cm^−1^ increased in intensity in the ICASO spectra compared to the R spectrum, while the bands at 1269/1228 cm^−1^ (in the ICASO spectra) are shifted from 1257/1234, 1244/1210, 1254/1218, and 1269/1221 cm^−1^ in the I, CA, SO, and R spectra, respectively.

The shifting of these bands and the variations in their intensities designate modifications in the structural environments of the specific groups, which might indicate the formation of new bonds between the wooden components and the imidazole, citric acid, and/or sorbitol.

**Figure 10:**
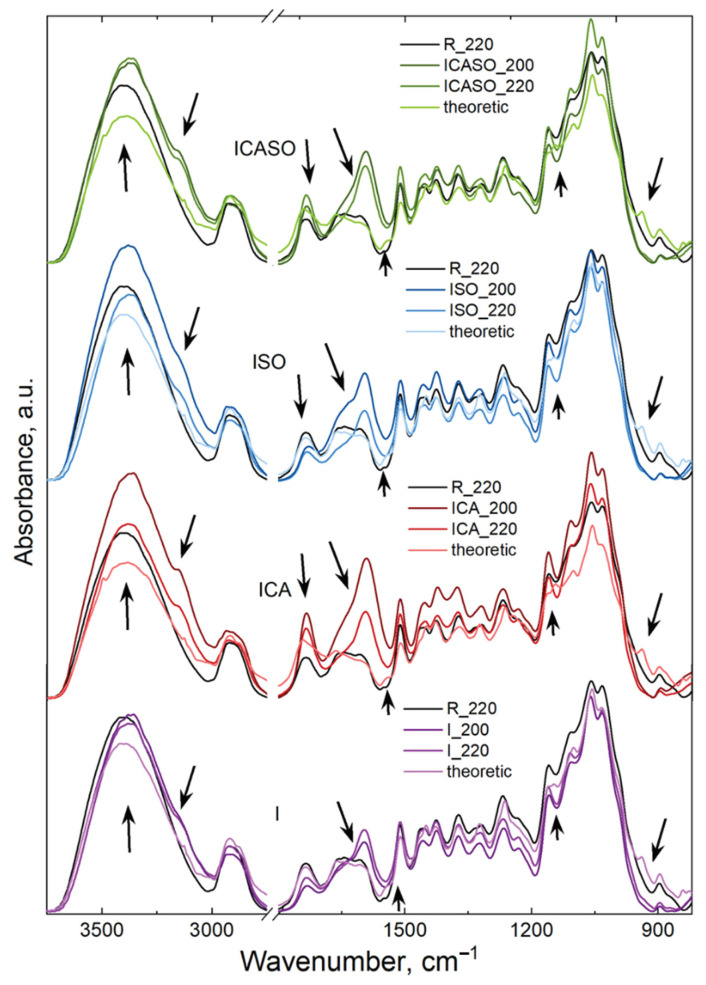
Infrared spectra of wood and pressed boards at 200 °C and 220 °C. The arrows indicate the modifications which are mentioned in the text.

To confirm the presence of interactions between the wood and chemicals used for the pressing of boards, the theoretical spectra were calculated according to the additivity law and are presented in Figure 10. For comparison, the reference wood spectrum (R) and experimental spectra of the pressed boards at 200 °C and 220 °C are also represented. 

The theoretic spectrum represents the case when no interactions take place between the board’s components (i.e., I and R, I, CA, and R, etc.) during pressing. As can be observed in all cases, the theoretic spectrum is very similar to the wood (R) spectrum, with very small differences observed for the bands at 3400 cm^−1^, assigned to different OH stretching vibrations; 1735 cm^−1^, assigned to C=O stretching vibrations in carbonyl and carboxyl groups; 1143 cm^−1^, assigned to C-N aliphatic stretching vibrations; and 938 cm^−1^, assigned to C-H stretching vibrations. These bands all belong to different adhesives used for the processing of particleboards.

On the other hand, the spectra of the pressed boards present differences (compared to the reference wood, as well as the theoretic spectrum): the differences are observed in the large band with a maximum at 3400 cm^−1^ and a shoulder at 3159 cm^−1^, assigned to stretching vibrations of different hydrogen bonds between the OH and HO groups, as well as between OH and NH/N groups; at 1734 cm^−1^, assigned to C=O stretching vibrations in carbonyl and carboxyl groups; and at 1591 cm^−1^, assigned to stretching vibrations of C=C in the aromatic ring of lignin but also to the stretching vibration of conjugated C-O groups and C=C from imidazolium ions (proton conjugating system) [69]. Detailed information about all differences appearing in the pressed boards are given above. Thus, the infrared spectra clearly indicate the presence of chemical interactions taking place between the wood and the chemicals used for the boards, as well as hydrogen bond interactions.

## 4. Conclusions

This study aimed to investigate a novel formaldehyde-free adhesive system for particleboards based on imidazole in combination with citric acid and sorbitol, fabricated at different pressing temperatures and using residuals from sawmill processing consisting of softwood particles. The hygroscopic and mechanical properties of the particleboards were tested.

For all tested chemical combinations, a pressing temperature of 220 °C led to the best results. Treatments utilizing imidazole provided the particleboards with excellent hygroscopic properties and good internal bonding strengths, while the addition of sorbitol reduced most properties. The best results were achieved with a combination of 14.4 wt% imidazole and 11.3 wt% citric acid. The thickness swelling after 24 h of water immersion was 6.3% and the internal bonding strength was 0.57 MPa. The cyclic accelerated ageing test showed extremely low thickness changes after 4 h of boiling in water when imidazole was used. However, the modulus of elasticity and modulus of rupture were very low, at 1.1 GPa and 3.3 Mpa, respectively. A possible explanation for these low values is an imidazole-promoted increased degradation of lignin in the outer layers of the board. Optimization of the pressing procedure, including chemical loading, pressing temperature, and time may improve these properties. FTIR spectroscopy can demonstrate chemical interactions between the wood and chemical reagents; however, the exact mechanism of action between imidazole and wood/citric acid is still uncertain.

The low bending properties led to none of the particleboards meeting the minimum requirements of particleboards for humid conditions, as specified in the EN 312 standard. Additionally, the use of imidazole as a reagent is not without concern, with risks to health reported. Therefore, further research is needed to understand the discrepancy between high internal bonding strength and low bending strength and how to improve these properties; additional chemical analysis is also required to understand how imidazole reacts with the other components and the types of residual chemicals left after pressing, as well as to investigate alternative chemicals for similar formaldehyde-free adhesive systems.

## Figures and Tables

**Table 1:** Material properties and pressing parameter for pressing of particleboards. Concentrations in wt% are target concentrations in the chemical-particle blend after drying. T = pressing temperature. Boards per group = 3.

Group ID	Imidazole [wt%]	Citric Acid [wt%]	Sorbitol [wt%]	Urea Form- Aldehyde [wt%]	Pressing Time [min]	T [°C]
I200	14.4	-	-	-	10	200
I220	14.4	-	-	-	10	220
CA200	-	11.3	-	-	10	200
CA220	-	11.3	-	-	10	220
ICA200	14.4	11.3	-	-	10	200
ICA220	14.4	11.3	-	-	10	220
ISO200	14.4	-	3.6	-	10	200
ISO220	14.4	-	3.6	-	10	220
ICASO200	14.4	7.6	2.4	-	10	200
ICASO220	14.4	7.6	2.4	-	10	220
UF180	-	-	-	12.5	4	180

**Table 2:** Specimen properties after pressing and after conditioning. EMC: equilibrium moisture content.

Treatment ID	Weight Loss after Pressing [%]	Density ± Std. Dev. at EMC [kg m^−3^]	EMC [%]	Thickness Swelling at EMC [%]
I200	6.0	711 ± 24	7.7	8.0
I220	8.8	747 ± 21	6.8	4.4
CA200	5.4	684 ± 34	8.2	6.9
CA220	7.7	705 ± 15	7.7	4.6
ICA200	9.9	700 ± 37	9.6	6.3
ICA220	14.3	679 ± 21	7.3	3.6
ISO200	6.9	679 ± 42	8.4	10.5
ISO220	8.9	725 ± 23	7.9	5.6
ICASO200	7.4	691 ± 31	8.7	6.2
ICASO220	11.5	704 ± 37	7.4	4.0
UF180	-	720 ± 38	7.7	2.1

## Data Availability

The data presented in this study are available on request from the corresponding author. All data are supported in the paper (with statistical variance).
